# Practical Departments

**Published:** 1895-10-12

**Authors:** 


### PRACTICAL DEPARTMENTS.

### DISINFECTION AND THE EXISTING DATA FOR ITS CHARITIES.—II. Methods of Disinfection.

The most satisfactory method of disinfecting is unquestionably by the use of steam, and the first point of importance is to determine the temperature and exposure which are necessary for absolute disinfection.

These conditions depend upon whether the steam is saturated or superheated. Steam can in either form be of any temperature whatever. The difference is that if a slightly colder object is introduced into saturated steam, condensation at once occurs, and without any fall of temperature the enormous amount of latent heat which has been absorbed in retaining the steam in the form of vapour is released and communicated to the adjacent object. On the other hand superheated steam (*i.e.*, steam which has been artificially heated beyond the temperature at which it boils or can condense under the existing pressure) has to be cooled by the slow process of conduction to its condensation or saturation point for the pressure in question before this latent heat is evolved. The usual method of applying superheat in this country is by the use of a jacket surrounding the disinfection chamber, and containing steam hotter than that used for the disinfection. The purely physical difference involves, obviously, a much longer exposure with superheated steam than with saturated, to heat the object to be disinfected to any temperature above saturation point of the steam, and, therefore, to complete its heating to the temperature of the superheated steam itself. This difficulty can be measured by thermometers, and some of its consequences were shown by Dr. Parsons' report of 1884, in which he obtained in no case a practically uniform distribution of heat throughout the disinfection chamber. But a still more important point, which was not discovered till some four years later, is that steam loses its special disinfectant value when it becomes superheated, and does not regain it until temperatures and exposures are reached such as are required for disinfection by hot air. The objection has been raised that with English superheated steam stoves "the difficulty has always been to bring the articles out dry, and as long as they come out with even half per cent. added moisture it is certain that the steam has never been entirely superheated." Neither the premiss nor the inference is correct. The difficulty named certainly exists in the case of mattresses or other thick objects, which by their bulk cool the steam to saturation temperature and keep it there sufficiently long to absorb a large quantity of water. In actual working, however, the lighter and more porous objects may actually come out drier than when they went in, even to the extent of being scorched. But supposing this had not been the case, the condensation of moisture in the object does not necessarily prevent the steam from being superheated. On the contrary, in some of the most conclusive experiments which have been made on the point, the objects, the exterior of which failed to be disinfected by superheated steam, were, in the same operation, thoroughly wetted in their interior by the steam which had been cooled to saturation and condensed, and the organisms there placed were entirely destroyed. The truth is that in those cases where superheated steam stoves give disinfection the action has arisen through the loss of the superheat and the failure of the object to reach the temperature of the steam either at any point of the operation, or, at least, until it had been exposed to saturated steam for a sufficiently long period. This is a most unsatisfactory state of things for several reasons. It is sometimes the practice, in order to accelerate the operation, that objects should be heated before the disinfection begins, and there is no evidence whatever that under such conditions, where they must frequently be incapable of reducing superheated steam to saturation, disinfection has actually occurred. Again, superheated steam, deriving some of its temperature from irregular currents instead of from internal heat due to its physical condition as vapour, is always irregular in temperature throughout the stove, and it is impossible to obtain from the pressure-gauge or otherwise the knowledge which is desirable of the temperature prevailing in the stove. Thus, no diagram reliably recording the temperature can be obtained, and this precaution, which is most valuable in securing the control by medical officers of the disinfections for which they are responsible, becomes either impracticable or illusory. All these disadvantages are naturally reflected in the practice of superheated steam disinfection. A much higher pressure of steam is used, a much less regular distribution of heat obtained, and both the process of penetration and that of drying take longer than with a good saturated steam stove. England is almost alone in continuing the use of this unscientific and disadvantageous process, and it is to be hoped that before long it will have disappeared from this country as it has from others.

A much more accurate method of comparison between various experiments is necessary to attain a reliable knowledge of the real temperature of true disinfection. That temperature may be defined in general terms as that which is necessary for the destruction of the most persistent known infectious matter. The comparison of experiments labours under this enormous difficulty: that very wide differences exist in the resistance of different specimens of the same organism, and disinfection may have been obtained from, say, anthrax in one set of experiments where a more resistant specimen of that bacillus would have survived. To some extent, also, even in saturated steam stoves the construction of the stove is of importance, as a stove which permits undue condensation on its walls may materially reduce the effective temperature at which the operation takes place, and necessitate a much longer exposure. The best means with which we are acquainted for this class of work is the saturated steam stove of Geneste and Herscher, known in this country under the name of Equifex, in which the requisite conditions above-mentioned are reproduced to a nicety. This form of stove is in general use in France, and has been adopted as the model in most other countries. This stove is interesting not only as representing the type of saturated steam stove which in practice has proved most satisfactory, but, still more, because it has been the subject of a very large mass of experiments, by means of which reliable data as to the temperature of true disinfection have been obtained. This temperature, as determined by a collation of experiments made under similar conditions in this apparatus, may be fixed at an exposure of 15 minutes to 112°C., which is more than obtained by a steam pressure in the stove of 10 lbs. per square inch.

At the same time cases frequently arise where an authority or institution is unable to afford the price of a stove working at this pressure. In such case there can be no doubt that for the ordinary non-spore-bearing contagia, such as, for instance, typhoid fever, a lower temperature may be taken. This temperature must be above boiling point, because, on the one hand, it is desirable to have as high a temperature as can be conveniently got, and, on the other hand, the presence of some pressure is necessary to enable the automatic record of temperature to be obtained by the recording pressure gauge. Probably some pressure between one and two pounds will be found most satisfactory and convenient. It must, however, be carefully borne in mind that the reduction of the temperature to this point is permitted purely for financial motives, and that the margin of strength will therefore be much less than that which is provided in the stoves working at higher pressure. It is, therefore, impossible with safety to accept a mere reproduction of the higher pressure stoves, made in thinner materials and without some of the more costly drying conveniences. In these stoves the pressure is limited by a mechanical valve. It is an indispensable condition of all stoves working at any pressure at all, and unprovided with a large margin of mechanical strength, that the pressure be limited by some other arrangement, of which the failure will not subject the stove to higher pressure than that for which it was built.

It must not, however, be forgotten that the simple process of boiling gives a valuable disinfection for many purposes. The condition of its being reliable is that the temperature obtained throughout the water is uniformly and constantly that of boiling point. This requires a special form of vessel, which should be simple, automatic in action, and entirely independent of any safety valve. With such an appliance the bed, table, and body linen of patients whose excreta in any form are liable to convey contagion can most simply and readily be disinfected. The process, applicable to all such contagia, is most especially applicable to that of tuberculosis, for the double reason that its simplicity permits disinfection to take place with the utmost promptitude, and that it is certain that the chief cause of tubercular infection is the dry sputa, &c., which could be dealt with in this way when fresh. If this simple means were generally used, the main cause of the communication of phthisis from person to person would be removed, and the dangers due to self-infection on the part of the patien twould be reduced.

